# Management of Inflammatory Bowel Disease during Pregnancy and Breastfeeding Varies Widely: A Need for Further Education

**DOI:** 10.1155/2016/6193275

**Published:** 2016-09-20

**Authors:** Vivian Wai-Mei Huang, Hsiu-Ju Chang, Karen Ivy Kroeker, Karen Jean Goodman, Kathleen M. Hegadoren, Levinus Albert Dieleman, Richard Neil Fedorak

**Affiliations:** ^1^Division of Gastroenterology, Faculty of Medicine, University of Alberta, Edmonton, AB, Canada; ^2^Faculty of Nursing, University of Alberta, Edmonton, AB, Canada

## Abstract

*Background. *Inflammatory bowel disease (IBD) affects patients in their young reproductive years. Women with IBD require maintenance therapies during pregnancy and breastfeeding. However, physician management of IBD during pregnancy and breastfeeding has not been well characterized.* Objective. *To characterize physician perceptions and management of IBD during pregnancy and breastfeeding.* Methods. *A cross-sectional survey of Canadian physicians who are involved in the care of women with IBD was conducted. The survey included multiple-choice and Likert scale questions about perceptions and practice patterns regarding the management of IBD during pregnancy and breastfeeding.* Results. *183 practicing physicians completed the questionnaire: 97/183 (53.0%) gastroenterologists; 75/183 (41.0%) general practitioners; and 11/183 (6.0%) other physicians. Almost half (87/183, 47.5%) of the physicians felt comfortable managing pregnant IBD patients. For specified IBD medications, proportions of physicians who indicated they would continue them during pregnancy were as follows: sulfasalazine, 47.4%; oral mesalamine, 67.0%; topical mesalamine, 70.3%; oral prednisone, 68.0%; topical prednisone, 78.0%; oral budesonide, 61.6%; topical budesonide, 75.0%; ciprofloxacin, 15.3%; metronidazole, 31.4%; azathioprine, 57.1%; methotrexate, 2.8%; infliximab, 55.6%; adalimumab, 78.1%. Similar proportions of physicians would continue these medications during breastfeeding. A higher proportion of gastroenterologists than nongastroenterologists indicated appropriate use of these IBD medications during pregnancy and breastfeeding.* Conclusions. *Physician management of IBD during pregnancy and breastfeeding varies widely. Relative to other physicians, responses of gastroenterologists more frequently reflected best practices pertaining to medications for control of IBD during pregnancy and breastfeeding. There is a need for further education regarding the management of IBD during pregnancy and breastfeeding.

## 1. Introduction

Inflammatory bowel disease (IBD) is a group of chronic inflammatory diseases that often affects patients in their young and reproductive period of life and thus requires lifelong medications. IBD is often managed using the “pyramid of treatment” approach. At the bottom level of the treatment pyramid are the sulfasalazine and 5-aminosalicylate (5-ASA) medications. However, some IBD patients require more aggressive medications such as immunosuppressants (azathioprine/6-mercaptopurine and methotrexate), corticosteroids (prednisone and budesonide), and biologics (anti-tumor necrosis factor alpha, e.g., infliximab and adalimumab). Each of these IBD medications is assigned an FDA (Food and Drug Administration) classification of safety for use during pregnancy and breastfeeding based on the available level of evidence from animal and human studies [1]. Despite recent studies showing that these IBD medications can be continued during pregnancy and breastfeeding with relatively low risk of congenital malformations, or adverse pregnancy and neonatal outcomes, patients and physicians continue to be concerned and unsure about the use of IBD medications during pregnancy among women with IBD [2–4].

IBD patients' reproductive wishes affect their treatment plans as shown by Zelinkova et al. who reported that a third of IBD patients who plan to have children require medication changes [5]. In addition, female IBD patients receive significantly less immunosuppressants compared to male IBD patients, although they may have higher disease activity, suggesting that there is a gender-specific difference in the therapeutic management of IBD patients [6]. This gender-specific difference in management may be related to inadequate knowledge or experience regarding the use of IBD medications during the reproductive period. For example, even among expert gastroenterologists who have published on the use of thiopurines in IBD, 89% would continue azathioprine until delivery, while 9% would never administer azathioprine during pregnancy [7]. In the Canadian health care system, the care of women with IBD involves various physician groups including general practitioners, internists, gastroenterologists, and obstetricians. General practitioners and internists often are the initial physicians for preconception and early pregnancy care.

The objective of this study was to assess the IBD-specific knowledge and use of IBD medications with respect to pregnancy and breastfeeding of practicing Canadian physicians who are involved in the care of women with IBD.

## 2. Methods

### 2.1. Study Design

This study was a cross-sectional survey assessing physician perception and practice patterns regarding the management of IBD during pregnancy and breastfeeding.

### 2.2. Settings and Participants

The study was conducted from the Inflammatory Bowel Disease Consultation and Research Clinic at the University of Alberta Hospital (Edmonton, Canada) between 2012 and 2013. In order to reach physicians who were involved in the care for women with IBD, the following physician groups were recruited: physicians who referred patients to the IBD clinic, physicians who attended the national Mentoring in IBD conference, physicians who attended the Gastroenterologists (GI) for General Practitioners (GP), a Northern Alberta educational event hosted by the University of Alberta on the management of GI diseases, and physician members of the Canadian Association of Gastroenterology (CAG). Referring physicians and physicians attending the conferences were given a study package consisting of an informed consent document that indicated consent would be implied by submitting the questionnaire, the study questionnaire, and a stamped self-addressed return envelope. Members of CAG were invited via directed email with a link to a web-based version of the questionnaire. After selecting the link, they were provided with the information letter and study questionnaire. Although physicians in training were at these conferences and were provided study packages, only practicing physicians were included in this analysis. All responses were collected anonymously, in order to encourage participation in the study. Since responses were anonymous, tracking of invited physicians and second recruitment attempts could not be conducted.

### 2.3. Data Sources and Variable Definitions

The study questionnaire (see Appendix in Supplementary Material available online at http://dx.doi.org/10.1155/2016/6193275) included questions on demographics (practice setting, scope of practice, proportion with IBD of patients in their practice, and number of pregnant IBD patients managed in the past year), comfort managing pregnant IBD patients, and use of medications to treat IBD (a) during pregnancy and (b) during breastfeeding. The questions on physician demographics were based on review of other survey-based studies on physician management of IBD [7–10]. The study questionnaire was reviewed by three IBD specialists (Karen Ivy Kroeker, Levinus Albert Dieleman, and Richard Neil Fedorak) and a clinical epidemiologist (Karen Jean Goodman) who provided feedback to increase validity (see Appendix for full questionnaire).

To characterize use of medications, physicians were asked the following for each medication category:

 Question # 17: Please indicate if you would stop the medication, continue the medication, continue adjusted, or are unsure, if your patient informed you she was trying to conceive, or that she was pregnant.
 Question # 18: Please indicate if you would stop the medication, continue the medication, continue adjusted, or are unsure, if your patient informed you she was breastfeeding.

Responses could be “stop,” “continue,” “continue adjusted,” or “unsure.”

The medication categories listed were sulfasalazine, mesalamine (5-aminosalicylate, 5-ASA) both oral and topical, prednisone (oral and topical), budesonide (oral and topical), ciprofloxacin, metronidazole, azathioprine/6-mercaptopurine, methotrexate, infliximab, and adalimumab. These were the most commonly used and nationally available medications used to treat IBD at the time of this survey study.

### 2.4. Methods Used to Reduce Bias

To enhance representativeness of participating physicians, all referring physicians to the University of Alberta IBD clinic, all physician members of CAG, and all physicians attending the conferences were invited to participate, without exclusionary criteria. Their responses were collected anonymously, to encourage participation and minimize selection bias due to hesitancy to participate because of identification. Measurement bias was minimized by using a mixture of detailed multiple choice and Likert-scale questions to accurately classify practice demographics and practice patterns.

### 2.5. Statistical Analysis

For continuous variables, medians and interquartile ranges (IQR) were tabulated, and medians were compared across subgroups using nonparametric Mann-Whitney and Kruskal Wallis tests. For categorical variables, frequency distributions of categories were tabulated, and differences in distributions were compared across subgroups using the Chi-square (*χ* ^2^) test. Certain categorical data were collapsed for comparisons among subgroups (see Appendix for full questionnaire, answers, and collapsed categories).

*Missing Responses*. As many respondents did not complete every medication-related questionnaire, for questions with missing responses, frequencies and percentages were calculated using the total number of responses for the specific question as the adjusted denominator. Statistical analyses was also conducted with nonresponse treated as incorrect answers and the total number of participants as the denominator.

*p* values for the null hypothesis of no difference are reported for the comparison of medians and frequency distributions. The statistical program SPSS version 21.0 (IBM Corp. Released 2012, IBM SPSS Statistics for Windows, Version 21.0, Armonk, NY) was used for all data analysis.

### 2.6. Ethical Considerations

This study and the study materials were approved by the Health Research Ethics Board (HREB) of the University of Alberta. Physicians were informed that their participation in this study was voluntary. Return of the completed questionnaires was considered informed consent, and results were kept anonymous.

## 3. Results

### 3.1. Characteristics of Study Population

In total, 120 study packages were handed out at Mentoring in IBD, 60 were handed out at GI for GP meeting, 250 were mailed to referring physicians, and e-mail invitations were sent to all CAG members (approximately 400 packages/invitations were given out). A total of 206 invitees returned completed questionnaires, 64 from Mentoring in IBD (53.3%), 20 from GI for GP (33.3%), 68 from referring physician mailouts (27.2%), and 54 from CAG e-invite responses (13.5%). After excluding the 23 physician-in-training questionnaires, 183 practicing Canadian physicians were included for analysis in this study. Sociodemographic characteristics and practice characteristics are shown in Table 1. More than two-thirds of the respondents were male physicians (69.4%). More than half of respondents identified themselves as practicing gastroenterologists (53.0%), while the rest were general practitioners (43.0%) or other specialists (6.0%). More than two-thirds (70.6%) of respondents reported working in a community setting; almost all the general practitioners (98.6%) identified themselves as working in the community, compared to only half of the gastroenterologists. Half the physicians surveyed (49.7%) reported that the proportion with IBD of patients in their practice was less than 10%; almost all the general practitioners fell in this category, compared to the other physician groups. Almost half the physicians surveyed (41.5%) had managed no pregnant IBD patients in the past year. Most general practitioners (72.0%) had managed no pregnant IBD patients in the past year, while most gastroenterologists had managed up to 10 pregnant IBD patients (69.1%) or more than 10 pregnant IBD patients (15.5%) in the past year.

### 3.2. Physician Perceptions

As shown in Table 2, more gastroenterologists than other specialists or general practitioners indicated that more than 50% of their female IBD patients of reproductive age inform them when they are trying to become pregnant. Similarly, more gastroenterologists than other specialists or general practitioners indicated that more than 50% of their female IBD patients of reproductive age inform them when they are pregnant.

### 3.3. Use of Sulfasalazine and 5-Aminosalicylates

As shown in Table 3, 47.4% of surveyed physicians indicated they would continue sulfasalazine treatment among pregnant women with IBD, while 67.0% would continue oral mesalamine and 70.3% would continue topical mesalamine. A higher proportion of gastroenterologists compared to other specialists and general practitioners would continue these medications during pregnancy. As shown in Table 4, 40.3% of surveyed physicians would continue sulfasalazine treatment among women with IBD who are breastfeeding, while 64.8% would continue oral mesalamine and 70.8% would continue topical mesalamine. Among gastroenterologists, a higher proportion would continue sulfasalazine during pregnancy than during breastfeeding.

### 3.4. Use of Corticosteroids

As shown in Table 3, 68.0% of surveyed physicians would continue oral prednisone and 78.8% would continue topical prednisone during pregnancy; 61.6% would continue oral budesonide and 75.0% would continue topical budesonide during pregnancy. Smaller proportions of general practitioners compared to gastroenterologists and other specialists indicated they would continue oral prednisone during pregnancy. As shown in Table 4, 73.3% of physicians would continue oral prednisone and 84.2% would continue topical prednisone during breastfeeding; 69.9% would continue oral budesonide and 79.8% would continue topical budesonide during breastfeeding. Similar proportions of physicians would continue oral and topical prednisone and budesonide during both pregnancy and breastfeeding.

### 3.5. Use of Antibiotics

As shown in Table 3, 15.3% of surveyed physicians would continue ciprofloxacin and 31.4% would continue metronidazole during pregnancy. A higher proportion of gastroenterologists compared to other specialists and general practitioners would continue ciprofloxacin during pregnancy. A higher proportion of general practitioners compared to other specialists and gastroenterologists would continue metronidazole during pregnancy. As shown in Table 4, 28.7% would continue ciprofloxacin and 35.8% would continue metronidazole during breastfeeding. It was not clear that the distribution of responses for the use of ciprofloxacin during breastfeeding differed across physician group beyond random variation, given a *p* value of 0.13.

### 3.6. Use of Immunosuppressants

As shown in Table 3, 57.1% of physicians surveyed indicated they would continue azathioprine during pregnancy; 2.8% would continue methotrexate during pregnancy. A larger proportion of gastroenterologists compared to other specialists and general practitioners indicated they would continue azathioprine/6-mercaptopurine during pregnancy. A higher proportion of gastroenterologists compared to other specialists and general practitioners indicated they would continue methotrexate during pregnancy. As shown in Table 4, 49.4% of physicians would continue azathioprine during breastfeeding; 8.5% would continue methotrexate during breastfeeding. A higher proportion of gastroenterologists indicated they would continue azathioprine/6-mercaptopurine and methotrexate during breastfeeding compared to other specialists and general practitioners.

### 3.7. Use of Biologics

As shown in Table 3, 55.6% of physicians surveyed would continue infliximab and 78.1% would continue adalimumab during pregnancy. A higher proportion of gastroenterologists compared to other specialists and general practitioners would continue these medications during pregnancy. As shown in Table 4, 52.3% of surveyed physicians would continue infliximab and 50.6% would continue adalimumab during breastfeeding. A higher proportion of gastroenterologists compared to other specialists and general practitioners would continue these medications during breastfeeding.

## 4. Discussion

Management of chronic disorders such as IBD in pregnancy and breastfeeding can be challenging because of concerns of the effects of the medications on pregnancy and neonatal outcomes. In the Canadian health care system, many physician groups of varying education and experience are involved in the care of women with IBD during preconception, pregnancy, and postpartum periods. Therefore, although certain groups may not see as many women with IBD who are pregnant as gastroenterologists may, these physician groups will at some point still be involved in the medical care of women with IBD during pregnancy. As the first point of contact, these physicians may have to address their patients' concerns and counsel them on medication use. Active IBD during pregnancy is associated with 4.48-fold increased risk of miscarriage, 2.66-fold increased risk of preterm birth, and 3.3-fold increased risk of low birth weight infants [11]; any case of inappropriate continuation or discontinuation of IBD medications may lead to an adverse pregnancy and neonatal outcome. Thus, physicians involved in the care of women with IBD should understand the indications and relative safety of various medications used to achieve and maintain IBD emission.

### 4.1. Sulfasalazine and 5-Aminosalicylates

Sulfasalazine and 5-aminosalicylate (5-ASA) medications are commonly used to treat ulcerative colitis (UC). Women with UC have a 2.19- (95% CI 1.25–3.97) fold risk of relapse during pregnancy and postpartum than nonpregnant women with IBD [12]. Overall, studies report no increased risk of poor pregnancy outcomes, or adverse neonatal outcomes with 5-ASA medications, and both sulfasalazine and mesalamine can be continued during pregnancy and breastfeeding; since sulfasalazine interferes with folate absorption, higher dose of folic acid than usual (2 mg/day) is recommended for supplementation [13–20]. In this study, a majority of general practitioners reported being unsure about whether to continue these medications in women with IBD. Since these medications can be prescribed and renewed by nongastroenterologists (in Canada), it is important that these physicians also be aware of the indication and safety of continuing these maintenance medications during pregnancy and breastfeeding.

### 4.2. Corticosteroids

Corticosteroids are used to treat severe flares of IBD, and although avoidance in the first trimester of pregnancy is recommended to decrease the potential risk of cleft palate, if required, corticosteroids can be continued or initiated in pregnancy to control active IBD [20]. As the highest levels appear in the breast milk in the first 4 hours after consumption, it is recommended that breastfeeding women who are taking corticosteroids “pump and dump” the breast milk during that 4-hour period after ingesting the medication [1, 20, 21]. In this study, a quarter of general practitioners reported they would stop oral corticosteroids during pregnancy, and half would stop corticosteroids during breastfeeding for women with IBD. Although the risks and benefits of using corticosteroid treatment for IBD during pregnancy and postpartum need to be made on an individual case basis, since general practitioners are actively involved in these time periods, they should be educated on the importance of early referral to the gastroenterologist to treat active IBD during pregnancy and on the importance of not stopping corticosteroids that were initiated by the gastroenterologist to treat active IBD.

### 4.3. Antibiotics: Metronidazole and Ciprofloxacin

Metronidazole (FDA Class B) and ciprofloxacin (FDA Class C) are commonly used to treat abscesses and fistulae in IBD. Animal studies showed carcinogenic effects from metronidazole, and early studies suggested a risk of cleft lip [18], but this has not been reported in humans [22]. If required, metronidazole can be used during pregnancy [21, 22]. Since metronidazole is excreted in breast milk and prolonged exposure to metronidazole is associated with potential toxicity, it is not recommended during breastfeeding, although if required it can be used with the recommendation of waiting 12 to 24 hours after receiving a dose of metronidazole before breastfeeding [20]. Because of the known risk of arthropathy with the use of ciprofloxacin, avoiding this medication during pregnancy is often recommended [20]; however, meta-analysis of human studies has reported no significant increase in major congenital anomalies, including musculoskeletal problems from the use of ciprofloxacin [23]. Ciprofloxacin is also detectable in the breast milk in small amounts [24], but short-term treatment can be used if indicated [20]. In summary, these antibiotics can be used during pregnancy and breastfeeding if absolutely required, although the best practice is to avoid ciprofloxacin. However, in this study, the majority of physicians surveyed would stop ciprofloxacin (72.2%) and metronidazole (54.9%) for IBD patients during pregnancy, and many would stop ciprofloxacin (49.4%) and metronidazole (41.5%) during breastfeeding. Improved physician knowledge regarding the indications and relative safety of using these medications if required to treat complications of active IBD during pregnancy and breastfeeding is needed.

### 4.4. Immunomodulators: Azathioprine, 6-Mercaptopurine, and Methotrexate

Although thiopurines are classified as FDA class D drugs because of teratogenicity reported in earlier animal studies, most studies report that the use of azathioprine/6-mercaptopurine during pregnancy in women with IBD is not associated with significant increased risk of preterm birth, low birth weight, neonatal adverse outcomes, or congenital abnormalities [1, 17, 25–31]. Expert opinion is to continue thiopurine use during pregnancy and breastfeeding to maintain remission of disease, especially since a flare is associated with risk of adverse outcomes of pregnancy [1, 20, 21, 31]. Based on a recent international survey, Peyrin-Biroulet et al. reported that 89% of gastroenterologists continue azathioprine until delivery and 9% never use azathioprine during pregnancy [7]. In this Canadian survey study, only 57.7% of physicians surveyed correctly responded that “during pregnancy azathioprine or 6-mercaptopurine can be continued,” and 26.3% indicated they would stop these medications during pregnancy. There was a clear difference in the use of these drugs among physician groups; 89.4% of gastroenterologists would continue azathioprine/6-MP during pregnancy compared to only 15.7% of general practitioners. Almost half of the general practitioners indicated they would stop azathioprine/6-mercaptopurine during pregnancy and breastfeeding. Stopping these maintenance medications is known to increase risk of disease flare; thus, it is important to educate physicians, in particular, general practitioners, about the relative safety of continuing AZA/6-MP during pregnancy and breastfeeding in IBD.

Methotrexate is another commonly used immunosuppressant for the treatment of IBD. However, it has a known risk of causing miscarriage and congenital malformations; therefore, it is contraindicated during conception and pregnancy and breastfeeding. Patients should discontinue methotrexate for at least 3 to 6 months prior to attempting to conceive [1, 20]. Methotrexate crosses into the breast milk [31] and, because of its toxicity, is contraindicated during breastfeeding [32, 33]. In this study, it is worrisome that 5.6% of surveyed physicians in this study would continue or were unsure about the use of methotrexate during pregnancy, and an even larger proportion, 30.7%, would continue or were unsure about the use of methotrexate during breastfeeding.

There is clearly a need for further education of physicians regarding the indications for the continued use or cessation of immunosuppressant medications such as azathioprine and methotrexate during preconception, pregnancy, and postpartum/breastfeeding time period.

### 4.5. Biologics: Infliximab and Adalimumab

Anti-tumour necrosis factor alpha (anti-TNF-*α*) inhibitors, such as infliximab and adalimumab, are commonly used to treat moderate to severe refractory IBD and fistulizing Crohn's disease. Initially infliximab and adalimumab use was reported in a few cases of pregnant women with IBD who did not experience any adverse effects [34–41]. It was previously recommended to stop infliximab and adalimumab at the onset of the third trimester to decrease the amount of placental transport [42–44]; however, more recent literature shows the placental transfer begins as early as 22–24 weeks of gestation, and thus some experts propose to stop anti-TNF therapy by 24 weeks to minimize fetal exposure (in women with sustained remission) [20, 45]. Since large observational studies, registry studies, and systematic reviews have shown safety for use of anti-TNF medications during pregnancy [44–49], it is recommended that if a woman with IBD requires anti-TNF therapy during pregnancy and postpartum, these medications may be continued [20, 45]. However, since the neonate may be exposed to anti-TNF medication and it has been detected in infant blood up to 6 months of age, it is recommended that infants exposed to anti-TNF therapy in utero avoid being given live vaccines until after 6 months of age [1, 20]. Studies have shown nil to minimal levels of infliximab and adalimumab in the breast milk and no significant adverse events have been reported in breastfeeding infants whose mothers take these drugs [38, 50–54]. It is thought that any detectable levels in the neonate after delivery may be due to placental transfer during pregnancy [54]. There was a clear lack of knowledge regarding the use of infliximab and adalimumab among physician respondents in this study. Almost 90% of general practitioners surveyed indicated they would stop or were unsure about the use of these biologics during pregnancy and breastfeeding. Even 10% of gastroenterologists indicated they would stop or were unsure about these medications. Thus, there is a need to improve physician knowledge regarding the use of biologics in the treatment of IBD during pregnancy and breastfeeding.

## 5. Limitations

Although this is the first published study assessing physician knowledge of the use of IBD medications during pregnancy and breastfeeding, there are a few limitations to the study. The study was designed for the Canadian health care system, in which multiple physician groups (general practitioners, internists, gastroenterologists, and obstetricians) are involved in the care of women with IBD. This study thus focused on practitioners of first point of contact for preconception or pregnant women with IBD (general practitioners, internists, and gastroenterologists), as obstetricians become involved later in pregnancy. We attempted to direct the study invitations to physicians who are involved in the care of women with IBD, by directing recruitment towards Mentoring in IBD attendees, GI for GP attendees, referring physicians to the IBD clinic, and members of CAG. It is possible that there were duplicated invitations as attendees of Mentoring in IBD and the other conferences may be members of CAG as well. However, it is assumed that they would not have responded by completing duplicate surveys. Response rate was low, as is often the case in survey studies. In particular, the response rate from invited general practitioners was only 27%, which may have been due to various factors including lack of interest, or loss of surveys. Future attempts at educational needs assessment surveys may include nonresponder options in order to know which physicians chose not to respond and for what reason. Nevertheless, assuming if the responders were general practitioners who had more interest in the topic than nonresponders, it is concerning regarding the lack of knowledge on these important topics.

A large proportion of respondents (mainly general practitioners) reported seeing fewer than 10 IBD patients each year or having managed no pregnant IBD patients within the previous year (and this may be a reason for deficits in knowledge). However, with an increasing prevalence of IBD in Canada, these physicians will eventually encounter the situation of a female patient with IBD who is preconception or pregnant. They will need to recognize the complex issues of pregnancy in a woman with IBD who may be on immunosuppressants or biologics and who requires medical counselling and appropriate referral to a gastroenterologist and an obstetrician for management of IBD in pregnancy. The results of this survey provide an assessment of a need to further educate physicians who may be involved in the care of women with IBD, so that they are equipped with appropriate knowledge for future encounters and future decision-making regarding the management of IBD during pregnancy and breastfeeding.

Another limitation of the study is that the survey asked physicians in general terms whether they would continue, stop, or were unsure about using the IBD medications during pregnancy or breastfeeding.

It is not known how accurately physicians report their practice patterns; they may be reporting what they believe to be the best practice rather than their actual practice. An attempt to minimize this bias was made by collecting responses anonymously. The attempt to increase response rate by collecting responses anonymously may have resulted in an additional limitation, as nonresponders could not be tracked and thus additional recruitment strategies could be conducted, and nonresponders could not be characterized. In addition, some degree of the apparent knowledge deficit and inappropriate use or cessation of medications may be due to inaccurate responses in completing the questionnaire (e.g., physicians in a hurry may have misread the question or accidently selected the wrong response). Additionally, in this complex topic, the risks and benefits of each medication may differ with different clinical scenarios and physicians may vary their practice depending on individual patient disease characteristics and concerns in a manner that was not captured by this survey. A limitation of the study is that although the CCPKnow score has been validated by the authors who developed the questionnaire, we had not validated the non-CCPKnow questions regarding medication use for reliability, test-retest, and internal consistency.

A future study of benefit may be a similar investigation of physician IBD medication use and practice patterns based on various clinical case scenarios, or through workshop discussions, to further investigate physician and patient risk/benefit analyses when deciding to continue or stop IBD medications during pregnancy and breastfeeding. Also, this study showed that more gastroenterologists seemed to use IBD medications appropriately compared to other specialists or general practitioners, and as there were more academics in the gastroenterologist group compared to the general practitioner group, the increased knowledge and appropriate IBD medication use may be considered a reflection of more specialized training and clinical experience. However, this would be significant, as in Canada and other health care systems it is the general physicians who tend to be the first point of contact for women who are preconception, pregnant, and breastfeeding, regardless of disease status. Thus, it is still important to assess the level of knowledge and the practice patterns of physicians who are involved in the care of women with IBD and who may influence medication use in these women.

## 6. Future Directions

Since the year after this survey study was completed, there has been a push towards increased awareness of this complex issue, and several specialized clinics focusing on the preconception, pregnancy, and postpartum management of IBD currently exist in Canada; these include clinics at the University of Calgary, the University of Alberta, and the University of Saskatchewan, with upcoming clinics at various other Canadian institutions. In 2013, interested clinicians and clinical researchers around Canada formed the Maternofetal Outcomes Research in IBD-Canadian Registry (MORe CaRe) in order to optimize the care of women with IBD during the preconception, pregnancy, and postpartum periods. In addition, in 2014, the Canadian Association of Gastroenterology Clinical Practice Guidelines committee initiated development of Clinical Practice Guidelines for the management of IBD and pregnancy, which will be extremely useful and practical for all physicians and health care professionals involved in the care of women with IBD. The consensus statements for the management of IBD in pregnancy have been published and are available for physicians to access [55]. The next step should be knowledge dissemination of these statements and educating clinicians, especially general practitioners, who in Canada and other similar health care systems are the first point of contact for women who are preconception or pregnant.

Since this field is very complex and expert opinion and guidelines constantly evolve based on the endless availability of new clinical outcomes and safety data regarding the use of IBD medications during pregnancy and breastfeeding, physicians who are involved in the care of women with IBD must continuously be able to recognize the issues surrounding the use of IBD medications during pregnancy and breastfeeding. First point of contact physicians such as general practitioners and internists must be able to recognize IBD activity during pregnancy in order to promptly refer their patient to the gastroenterologist for medication and IBD management, and they must be aware of the risks and benefits of continuing or stopping the various IBD medications before they provide any medication advice to their patients.

Future studies should be conducted assessing the knowledge regarding IBD medication use during pregnancy and breastfeeding among other health care professionals who are involved in the care of pregnant women with IBD, such as obstetricians, midwives, nurses, and pharmacists. Future studies should also assess the optimal method of knowledge translation of this very complex topic to these various health care professionals so that the entire team, including the patient, is on the same care pathway.

## 7. Conclusion

Physicians have variable knowledge regarding the use of IBD medications during pregnancy and breastfeeding among women with IBD. Gastroenterologists demonstrated a high level of knowledge in accordance with best practice regarding the use of IBD medications during pregnancy and breastfeeding. However, knowledge deficits of nonspecialty physicians (e.g., general practitioners) regarding medications used to treat IBD in pregnant and breastfeeding women should be addressed with targeted educational activities, as these physicians are actively involved in the care of women with IBD during the preconception, pregnancy, and peripartum breastfeeding periods. Further studies addressing the knowledge and practice of providers of health care to women with IBD, including obstetricians, maternal fetal medicine specialists, nurses, midwives, and pharmacists, should be conducted to identify potential knowledge deficits and targets for educational activities.

## Supplementary Material

The appendix shows the full questionnaire given to the study participants. For some questions, responses were reclassified and collapsed into fewer response groups for statistical analyses.

## Figures and Tables

**Table 1:** Characteristics of practicing Canadian physicians surveyed regarding the management of IBD during pregnancy and breastfeeding.

	All physicians		Gastroenterologists		Other specialists		General practitioners		*p* value
	*n*/*N* ^*∗*^	%^*∗∗*^	*n*/*N* ^*∗*^	%^*∗∗*^	*n*/*N* ^*∗*^	%^*∗∗*^	*n*/*N* ^*∗*^	%^*∗∗*^	
*Gender*									
Male	127	69.4	76/97	78.4	9/11	81.8	47/84	56.0	0.005
Female	56	30.6	21/97	21.6	2/11	18.2	37/84	44.0	
*Training status *									
Gastroenterologist	97	53.0	/	/	/	/	/	/	
General practitioner	75	41.0	/	/	/	/	/	/	
Other	11	6.0	/	/	/	/	/	/	
*Years in practice *									
<5 years	27	14.8	20/97	20.6	1/11	9.1	6/84	8.0	0.001
5 to 10 years	26	14.2	22/97	22.7	1/11	9.1	3/84	4.0	
11 to 20 years	47	25.7	19/97	19.6	3/11	27.3	25/84	33.3	
>20 years	83	45.4	36/97	37.1	6/11	54.5	41/84	54.7	
*Population of city* (*n* = 131)^*∗*^									
<100,000	46	35.1	7/47	14.9	4/9	44.4	35/75	46.7	0.007
100,000 to 499,999	28	21.4	12/47	25.5	1/9	11.1	15/75	20.0	
>500,000	57	43.5	28/47	59.6	4/9	44.4	25/75	33.3	
*Practice setting* (*n* = 180)^*∗*^									
Community	127	70.6	48/96	50.0	7/11	63.6	72/84	98.6	<0.001
Academic	53	29.4	48/96	50.0	4/11	36.4	1/73	1.4	
*Percentage of patients with IBD in practice *									
<10%	91	49.7	12/97	12.4	6/11	54.5	73/84	97.3	<0.001
10–24%	51	27.9	46/97	47.4	3/11	27.3	2/84	2.7	
25–50%	27	14.8	26/97	28.8	1/11	9.1	0	0	
>50%	14	7.7	13/97	13.4	1/11	9.1	0	0	
*Number of IBD patients seen each year *									
0 to 9	33	18.0	1/97	1.0	3/11	27.3	29/84	38.7	<0.001
10 to 50	66	36.1	18/97	18.6	5/11	45.5	43/84	57.4	
51 to 100	18	9.8	15/97	15.5	1/11	9.1	2/84	2.7	
101 to 150	18	9.8	18/97	18.6	0	0	0	0	
More than 150	48	26.2	45/97	46.4	2/11	18.2	1/84	1.3	
*Number of pregnant IBD patients managed in past year *									
None	76	41.5	15/97	15.5	7/11	63.6	54/84	72.0	<0.001
Up to 10	70	38.3	67/97	69.1	1/11	9.1	2/84	2.7	
11 and more	37	20.2	15/97	15.5	3/11	27.3	19/84	25.3	

^*∗*^ *n* = number of responses falling into the response category; *N* = number of physicians who answered the question.

^*∗∗*^Percentages are calculated using the number of physicians who selected the response category as the numerator and the total number of physicians of that training status who provided a response to the question as the denominator.

GI: gastroenterologist, GP: general practitioner, and other: other specialists (general internists and surgeons).

IBD: inflammatory bowel disease.

**Table 2:** Physician perception of the percentage of their female IBD patients reporting pregnancy or pregnancy intentions.

	<10%		10–24%		25–50%		>50%		*p* value
	*n*/*N* ^*∗*^	%^*∗∗*^	*n*/*N* ^*∗*^	%^*∗∗*^	*n*/*N* ^*∗*^	%^*∗∗*^	*n*/*N* ^*∗*^	%^*∗∗*^	
*What percentage of your female IBD patients of reproductive age inform you when they are trying to become pregnant?*									
GI	13/95	13.7	16/95	(16.8%)	15/95	15.8	51/75	53.7	<0.001
GP	44/73	60.3	9/73	(12.3%)	8/73	11.0	12/73	16.4	
Other	5/11	45.5	1/11	(9.1%)	1/11	9.1	4/11	36.4	

All physicians	62/179	34.6	26/179	14.5	24/179	13.4	67/179	37.4

*What percentage of your female IBD patients in your practice inform you when they are pregnant?*									
GI	7/96	7.3	2/96	2.1	3/96	3.1	84/96	87.5	0.001
GP	24/73	32.9	3/73	4.1	4/73	5.5	42/73	57.5	
Other	3/11	27.3	0/11	0.0	1/11	9.1	7/11	63.6	

All physicians	34/180	18.9	5/180	2.8	8/180	4.4	133/180	73.9

^*∗*^ *n* = number of responses falling into the response category; *N* = number of physicians who answered the question.

^*∗∗*^Percentages are calculated using the number of physicians who selected the response category as the numerator and the total number of physicians of that training status who provided a response to the question as the denominator.

GI: gastroenterologist, GP: general practitioner, and other: other specialists (general internists and surgeons).

IBD: inflammatory bowel disease.

**Table 3:** Continuation of commonly used IBD medications for women with IBD during pregnancy by physician training status: a survey of practicing Canadian physicians.

	All physicians		Gastroenterologists		Other specialists		General practitioners		*p* value
	*n*/*N* ^*∗*^	%^*∗∗*^	*n*/*N* ^*∗*^	%^*∗∗*^	*n*/*N* ^*∗*^	%^*∗∗*^	*n*/*N* ^*∗*^	%^*∗∗*^	
Sulfasalazine and mesalamine									
Sulfasalazine									
Continue	**81/171**	**47.4**	**60/91**	**65.9**	**3/9**	**33.3**	**18/71**	**25.4**	<0.001
Stop	39/171	22.8	23/91	25.3	3/9	33.3	13/71	18.3	
Unsure	51/171	29.8	8/91	8.8	3/9	33.3	40/71	56.3	
Mesalamine, oral									
Continue	**118/176**	**67.0**	**95/96**	**99.0**	**5/10**	**50.0**	**18/70**	**25.7**	<0.001
Stop	12/176	6.8	1/96	1.0	0/10	0	11/70	15.7	
Unsure	46/176	26.1	0/96	0	5/10	50.0	41/70	58.6	
Mesalamine, topical									
Continue	**121/172**	**70.3**	**91/94**	**96.8**	**5/10**	**50.0**	**25/68**	**36.8**	<0.001
Stop	7/172	4.1	3/94	3.2	0/10	0	4/68	5.9	
Unsure	44/172	25.6	0/94	0	5/10	50.0	39/68	57.4	
Steroids									
Prednisone, oral									
Continue	119/175	68.0	81/95	85.3	8/11	72.7	30/69	43.5	<0.001
Stop	32/175	18.3	14/95	14.7	1/11	9.1	17/69	24.6	
Unsure	24/175	13.7	0/95	0	2/11	18.2	22/69	31.9	
Prednisone, topical									
Continue	141/179	78.8	85/96	88.5	8/11	72.7	48/72	66.7	<0.001
Stop	16/179	8.9	9/96	9.4	1/11	9.1	9/72	8.3	
Unsure	22/179	12.3	2/96	2.1	2/11	18.2	18/72	25.0	
Budesonide, oral									
Continue	106/172	61.6	75/93	80.6	4/10	40.0	27/69	39.1	<0.001
Stop	27/172	15.7	12/93	12.9	2/10	20.0	13/69	18.8	
Unsure	39/172	22.7	6/93	6.5	4/10	40.0	29/69	42.0	
Budesonide, topical									
Continue	129/172	75.0	82/92	89.1	6/10	60.0	41/70	58.6	<0.001
Stop	12/172	7.0	7/92	7.6	1/10	10.0	4/70	5.7	
Unsure	31/172	18.0	3/92	3.3	3/10	30.0	25/70	35.7	
Antibiotics									
Ciprofloxacin									
Continue	27/176	15.3	21/95	22.1	1/10	10.0	5/71	7.0	0.016
Stop	127/176	72.2	66/95	69.5	9/10	90.0	52/71	73.2	
Unsure	22/176	12.5	8/95	8.4	0/10	0	14/71	19.7	
Metronidazole									
Continue	55/175	31.4	27/95	28.4	3/11	27.3	25/69	36.2	0.003
Stop	96/175	54.9	61/95	64.2	8/11	72.7	27/69	39.1	
Unsure	24/175	13.7	7/95	7.4	0/11	0	17/69	24.6	
Immunosuppressants									
Azathioprine/6-mercaptopurine									
Continue	**100/175**	**57.1**	**84/94**	**89.4**	**5/11**	**45.5**	**11/70**	**15.7**	<0.001
Stop	46/175	26.3	8/94	8.5	4/11	36.4	34/70	48.6	
Unsure	29/175	16.6	2/94	2.1	2/11	18.2	25/70	35.7	
Methotrexate									
Continue	5/177	2.8	4/96	4.2	0/11	0	1/70	1.4	0.039
Stop	**159/177**	**89.8**	**90/96**	**93.8**	**10/11**	**90.9**	**59/70**	**84.3**	
Unsure	13/177	2.8	2/96	2.1	1/11	9.1	10/70	14.3	
Biologics									
Infliximab									
Continue	**99/178**	**55.6**	**87/96**	**90.6**	**4/11**	**36.4**	**8/71**	**11.3**	<0.001
Stop	39/178	21.9	4/96	4.2	5/11	45.5	30/71	42.3	
Unsure	40/178	22.5	5/96	5.2	2/11	18.2	33/71	46.5	
Adalimumab									
Continue	**96/177**	**54.2**	**84/95**	**88.4**	**4/11**	**36.4**	**8/71**	**11.3**	<0.001
Stop	37/177	20.9	3/95	3.2	5/11	45.5	29/71	40.8	
Unsure	44/177	24.9	8/95	8.4	2/11	18.2	34/71	47.9	

^*∗*^ *n* = number of responses falling into the response category; *N* = number of physicians who answered the question.

^*∗∗*^Percentages are calculated using the number of physicians who selected the category response as the numerator and the total number of physicians of that training status who provided a response as the denominator.

GI: gastroenterologist, GP: general practitioner, and other: other specialists (general internists and surgeons).

IBD: inflammatory bowel disease.

Bolded responses are best practice as recommended by expert opinion and guidelines.

**Table 4:** Continuation of commonly used IBD medications for women with IBD during breastfeeding by physician training status: a survey of practicing Canadian physicians.

	All physicians		Gastroenterologists		Other specialists		General practitioners		*p* value
	*n*/*N* ^*∗*^	%^*∗∗*^	*n*/*N* ^*∗*^	%^*∗∗*^	*n*/*N* ^*∗*^	%^*∗∗*^	*n*/*N* ^*∗*^	%^*∗∗*^	
Sulfasalazine and mesalamine									
Sulfasalazine									
Continue	**71/176**	**40.3**	**46/94**	**48.9**	**3/10**	**30.0**	**22/72**	**30.6**	<0.001
Stop	45/176	25.6	31/94	33.0	4/10	40.0	10/72	13.9	
Unsure	60/176	34.1	17/94	18.1	3/10	30.0	40/72	55.6	
Mesalamine, oral									
Continue	**116/179**	**64.8**	**91/96**	**94.8**	**5/11**	**45.5**	**20/72**	**27.8**	<0.001
Stop	11/179	6.1	2/96	2.1	2/11	18.2	7/72	9.7	
Unsure	52/179	29.1	3/96	3.1	4/11	36.4	45/72	62.5	
Mesalamine, topical									
Continue	**126/178**	**70.8**	**92/96**	**95.8**	**5/10**	**50.0**	**29/72**	**40.3**	<0.001
Stop	7/178	3.9	2/96	2.1	1/10	10.0	4/72	5.6	
Unsure	45/178	25.3	2/96	2.1	4/10	40.0	39/72	54.2	
Steroids									
Prednisone, oral									
Continue	132/180	73.3	88/97	90.7	8/11	72.7	36/72	50.0	<0.001
Stop	21/180	11.7	8/97	8.2	1/11	9.1	12/72	16.7	
Unsure	27/180	15	1/97	1.0	2/11	18.2	24/72	33.3	
Prednisone, topical									
Continue	149/177	84.2	93/96	96.9	9/10	90.0	47/71	66.2	<0.001
Stop	6/177	3.4	3/96	3.1	0/10	0	3/71	4.2	
Unsure	22/177	12.4	0/96	0	1/10	10.0	21/71	29.6	
Budesonide, oral									
Continue	121/173	69.9	84/94	89.4	6/11	54.5	31/68	45.6	<0.001
Stop	17/173	9.8	5/94	5.3	2/11	18.2	10/68	14.7	
Unsure	35/173	20.2	5/94	5.3	3/11	27.3	27/68	39.7	
Budesonide, topical									
Continue	138/173	79.8	87/94	92.6	7/10	70.0	44/69	63.8	<0.001
Stop	11/173	6.4	5/94	5.3	1/10	10.0	5/69	7.2	
Unsure	24/173	13.9	2/94	2.1	2/10	20.0	20/69	29.0	
Antibiotics									
Ciprofloxacin									
Continue	51/178	28.7	32/97	33.0	2/11	18.2	17/70	24.3	0.133
Stop	88/178	49.4	44/97	45.4	9/11	81.8	35/70	50.0	
Unsure	39/178	21.9	21/97	21.6	0/11	0	18/70	25.7	
Metronidazole									
Continue	63/176	35.8	30/96	31.3	3/11	27.3	30/69	43.5	0.014
Stop	73/176	41.5	46/96	47.9	8/11	72.7	19/69	27.5	
Unsure	40/176	22.7	20/96	20.8	0/11	0	20/69	29.0	
Immunosuppressants									
Azathioprine/6-mercaptopurine									
Continue	**87/176**	**49.4**	**76/95**	**80.0**	**5/11**	**45.5**	**6/70**	**8.6**	<0.001
Stop	47/176	26.7	11/95	11.6	4/11	36.4	32/70	45.7	
Unsure	42/176	23.9	8/95	8.4	2/11	18.2	32/70	45.7	
Methotrexate									
Continue	15/176	8.5	13/95	13.7	0/11	0	2/70	2.9	0.004
Stop	**122/176**	**69.3**	**68/95**	**71.6**	**10/11**	**90.9**	**44/70**	**62.9**	
Unsure	39/176	22.2	14/95	14.7	1/11	9.1	24/70	34.3	
Biologics									
Infliximab									
Continue	**92/176**	**52.3**	**83/95**	**87.4**	**4/11**	**36.4**	**5/70**	**7.1**	<0.001
Stop	34/176	19.3	5/95	5.3	5/11	45.5	24/70	34.3	
Unsure	50/176	28.4	7/95	7.4	2/11	18.2	41/70	58.6	
Adalimumab									
Continue	**89/176**	**50.6**	**80/95**	**84.2**	**4/11**	**36.4**	**5/70**	**7.1**	<0.001
Stop	33/176	18.2	5/95	5.3	5/11	45.5	23/70	32.9	
Unsure	54/176	30.7	10/95	10.5	2/11	18.2	42/70	60.0	

^*∗*^ *n* = number of responses.

^*∗∗*^Percentages are calculated using the number of physicians who selected the category response as the numerator and the total number of physicians of that training status who provided a response as the denominator.

Bolded responses are best practice as recommended by expert opinion and guidelines.
