# Supplementary material for: Management of Inflammatory Bowel Disease during Pregnancy and Breastfeeding Varies Widely: A Need for Further Education
Source: Can J Gastroenterol Hepatol. 2016 Sep 20;2016:6193275. doi: 10.1155/2016/6193275 (PMC5048030; doi:10.1155/2016/6193275)
Supplement: Supplementary file 1 — The appendix shows the full questionnaire given to the study participants. For some questions, responses were reclassified and collapsed into fewer response groups for statistical analyses. [file 6193275.f1.pdf]

APPENDIX: Physician questionnaire

1) What is your gender?

- ☐ 0 Male
- ☐ 1 Female

2) Please indicate which of the following best describes you:

- ☐ 0 Gastroenterology trainee
- ☐ 1 Surgical trainee
- ☐ 2 General Internist
- ☐ 3 Gastroenterologist
- ☐ 4 Surgeon
- ☐ 5 General Practitioner
- ☐ 6 Other healthcare professional
  Specify___________________

If you are a trainee, please skip # 3 – 5 and go to # 6.

3) How many years have you been in practice?

- ☐ 0 < 5 years
- ☐ 1 5 to 10 years
- ☐ 2 11 to 20 years
- ☐ 3 >20 years

4) What type of practice setting do you work in (>50%)?

- ☐ 0 clinical, community hospital
- ☐ 1 private outpatient clinic
- ☐ 2 clinical, academic hospital
- ☐ 3 research, academic hospital

5) What type of practice do you have?

- ☐ 0 Gastroenterology focus
- ☐ 1 Gastroenterology/Hepatology
- ☐ 2 Hepatobilary focus
- ☐ 3 General Medicine
- ☐ 4 General Surgery
- ☐ 5 Family practice
- ☐ 6 Other: specify ________________

6) Approximately what is the population of the town/city that you practice in?

- ☐ 0 <1,000
- ☐ 1 1,000 to 2,499
- ☐ 2 2,500 to 4,999
- ☐ 3 5,000 to 9,999
- ☐ 4 10,000 to 49,999
- ☐ 5 50,000 to 99,999
- ☐ 6 100,000 to 499,999
- ☐ 7 >500,000

7) What percentage of your patients have IBD?

- ☐ 0 <10%
- ☐ 1 10-24%
- ☐ 2 25 – 50%
- ☐ 3 >50%

APPENDIX: Physician questionnaire

8) How many IBD patients do you see each year?

- ☐$_0$ 0 - 9
- ☐$_1$ 10 - 50
- ☐$_2$ 51 - 100
- ☐$_3$ 100 - 150
- ☐$_4$ > 150

9) How many pregnant IBD patients have you managed in the past year?

- ☐$_0$ None
- ☐$_1$ up to 10
- ☐$_2$ 10 – 20
- ☐$_3$ >20

10) How do you feel about your IBD knowledge in general?

- ☐$_0$ Inadequate
- ☐$_1$ Enough to get by
- ☐$_2$ Very good

11) Do you feel comfortable managing pregnant IBD patients?

- ☐$_1$ Not comfortable at all
- ☐$_2$ Not comfortable
- ☐$_3$ Somewhat
- ☐$_4$ Fairly comfortable
- ☐$_5$ Very comfortable

12) With a female IBD patient of reproductive age, do you routinely bring up the topic of family planning?

- ☐$_0$ No
- ☐$_1$ Yes

13) What percentage of your female IBD patients of reproductive age inform you when they are trying to become pregnant?

- ☐$_0$ <10%
- ☐$_1$ 10-24%
- ☐$_2$ 25 – 50%
- ☐$_3$ >50%

14) With a female IBD patient of reproductive age who has been trying to conceive for more than 1 year, would you refer to a fertility specialist or gynaecologist?

- ☐$_0$ No
- ☐$_1$ Yes

15) What percentage of your female IBD patients in your practice inform you when they are pregnant?

- ☐$_0$ <10%
- ☐$_1$ 10-24%
- ☐$_2$ 25 – 50%
- ☐$_3$ >50%

16) Do you routinely refer your pregnant IBD patients to an obstetrician?

- ☐$_0$ No
- ☐$_1$ Yes

APPENDIX: Physician questionnaire

17) Please indicate if you would stop the medication, continue the medication, or are unsure, if your patient informed you she was **trying to conceive, or that she was pregnant.**

Medication	STOP	CONTINUE unchanged	CONTINUE at an adjusted dose or frequency	UNSURE
Sulfasalazine				
Mesalamine (oral)				
Mesalamine (topical)				
Prednisone (oral)				
Prednisone (topical)				
Budesonide/Entocort (oral)				
Budesonide/Entocort (topical)				
Azathioprine/6-MP				
Ciprofloxacin				
Metronidazole				
Methotrexate				
Biologics (Infliximab)				
Biologics (Adalimumab)				

18) Please indicate if you would stop the medication, continue the medication, or are unsure, if your patient informed you she was **breastfeeding.**

Medication	STOP	CONTINUE unchanged	CONTINUE at an adjusted dose or frequency	UNSURE
Sulfasalazine				
Mesalamine (oral)				
Mesalamine (topical)				
Prednisone (oral)				
Prednisone (topical)				
Budesonide/Entocort (oral)				
Budesonide/Entocort (topical)				
Azathioprine/6-MP				
Ciprofloxacin				
Metronidazole				
Methotrexate				
Biologics (Infliximab)				
Biologics (Adalimumab)				

APPENDIX: Physician questionnaire

Reclassification of responses for statistical analyses

- Question 2) In classifying training status, any responses other than “Gastroenterologist” or “General Practitioner” were combined into a single category “Other specialists” (there were only general internists and surgeons).
- Question 4) Type of practice setting was collapsed so that “clinical, community hospital” and “private outpatient clinic” were combined as “community”, and “clinical, academic hospital and “research, academic hospital” were combined “academic”.
- Question 6) Population of city was collapsed so that any response to population up to 99,999 was combined into “0 to 99,999”, while “100,000 to 499,999” and “>500,000” remained the same.
- Question 7) The number of pregnant IBD patients managed in the past year was collapsed so that “10 – 20” and “>20” were grouped into “11 or more”.
- Question 11) Regarding degree of comfort managing pregnant IBD patients, “not comfortable at all” and “not comfortable” were grouped as “not comfortable”, and “fairly comfortable” and “very comfortable” were grouped as “comfortable”.
- Question 17) and 18) Regarding medication use during pregnancy and breast feeding, responses of “continue” and “continue adjusted” were analyzed together as “continue”
